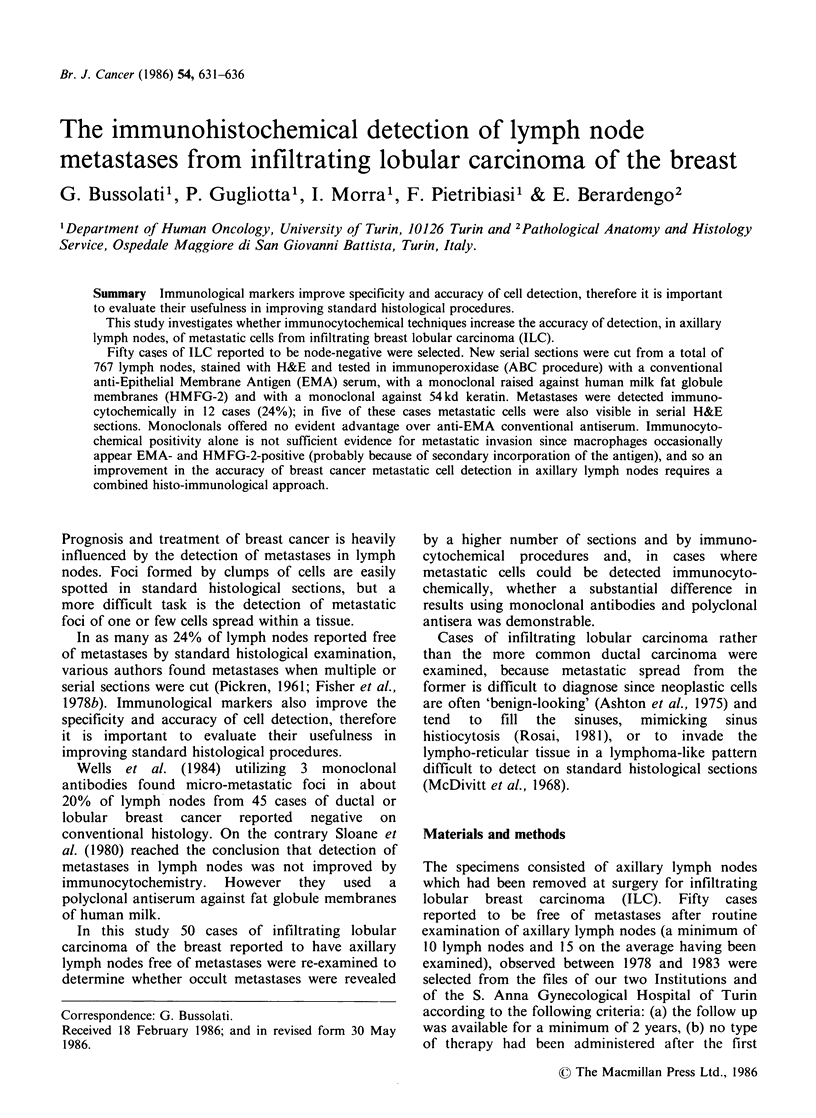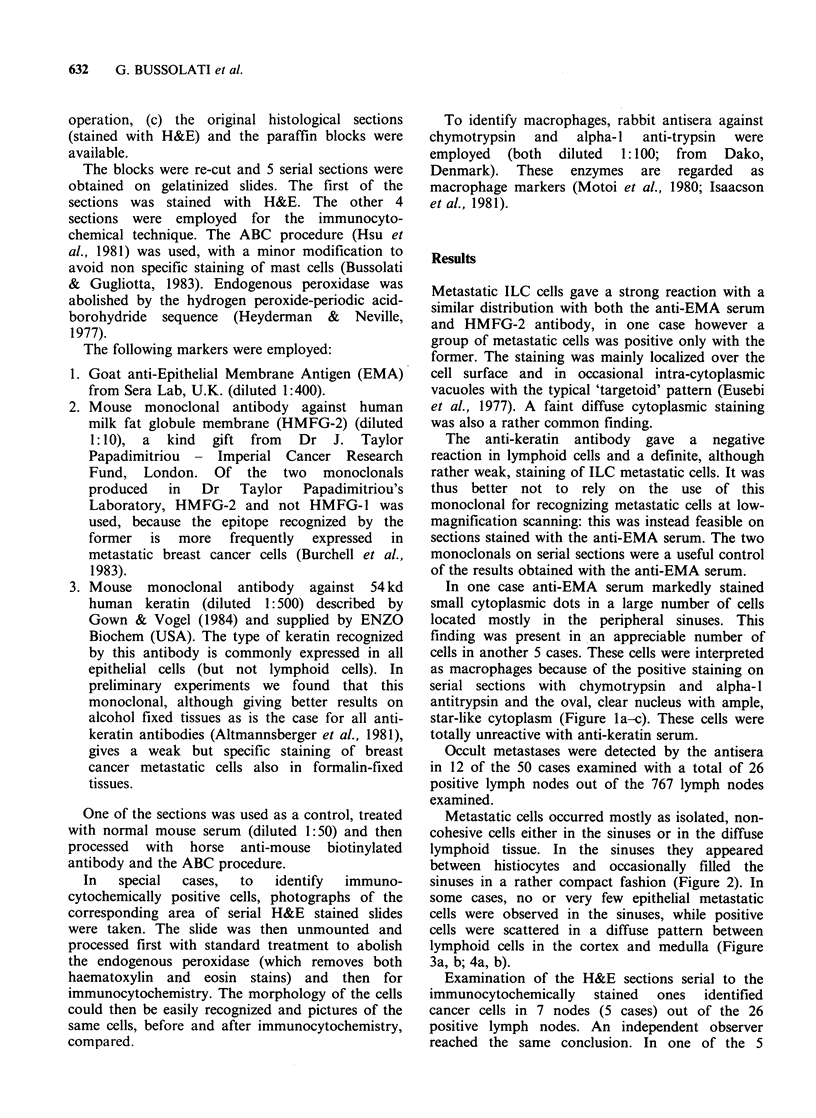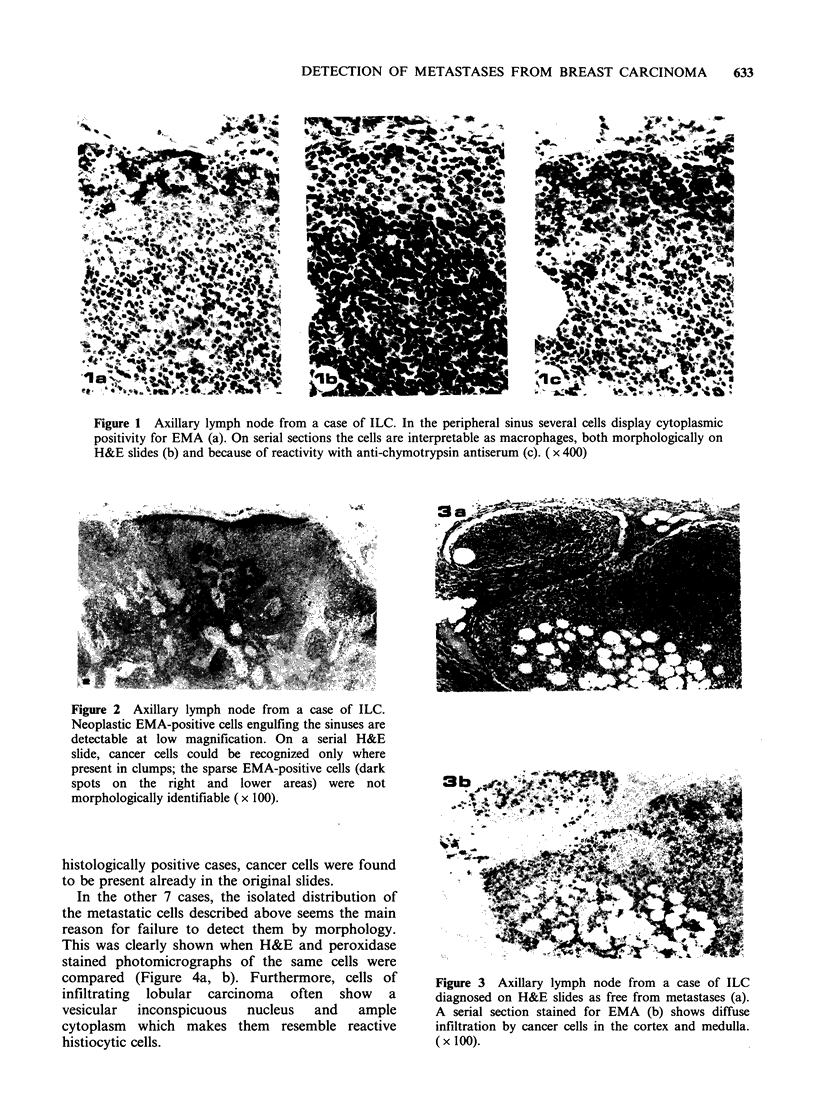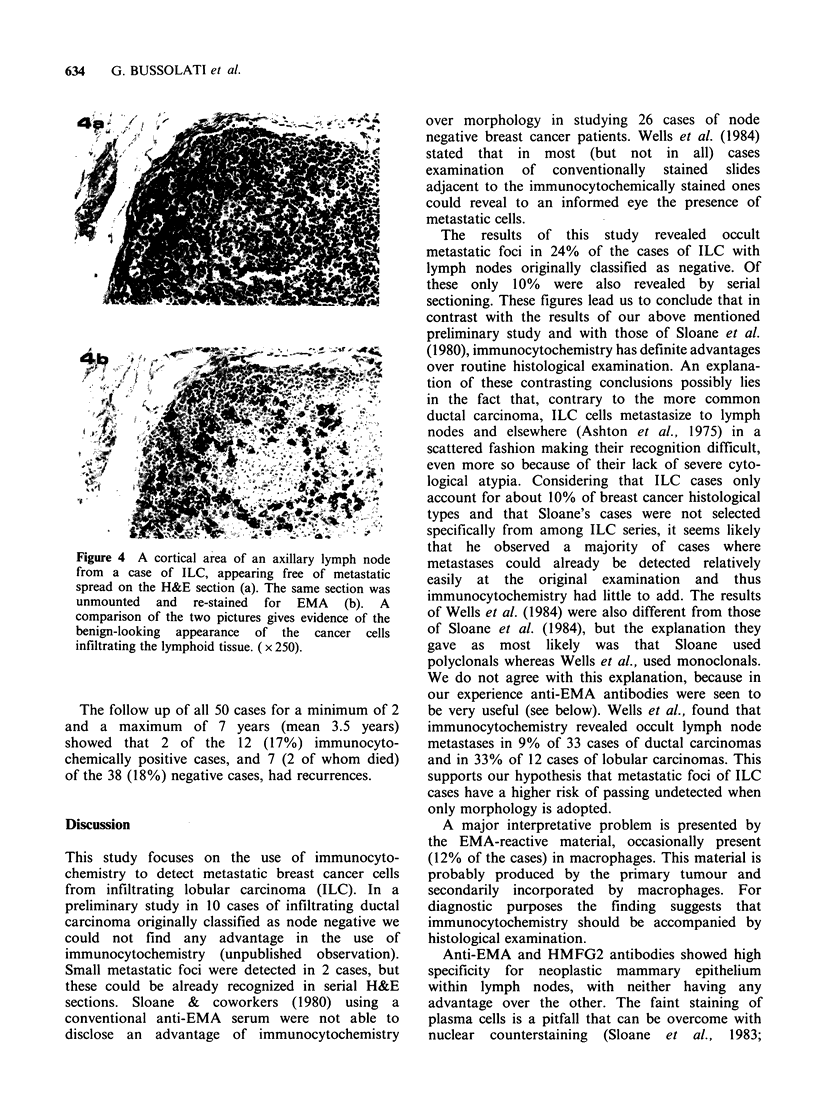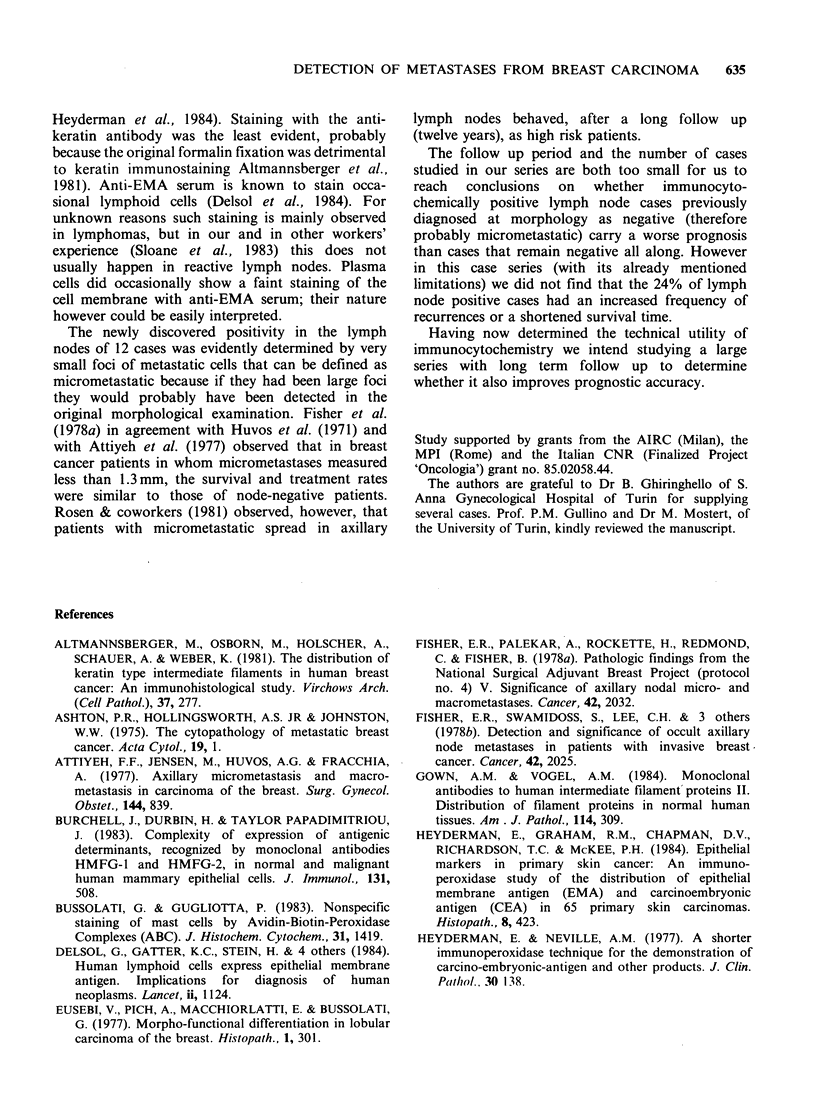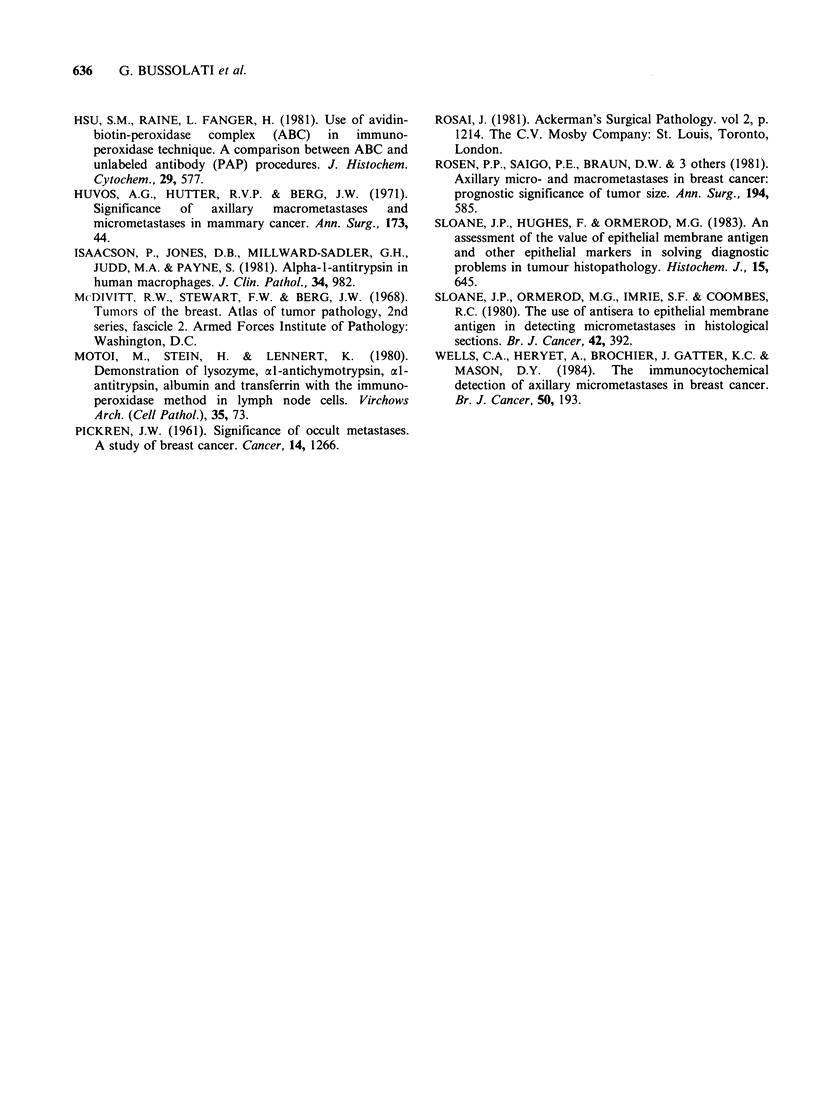# The immunohistochemical detection of lymph node metastases from infiltrating lobular carcinoma of the breast.

**DOI:** 10.1038/bjc.1986.219

**Published:** 1986-10

**Authors:** G. Bussolati, P. Gugliotta, I. Morra, F. Pietribiasi, E. Berardengo

## Abstract

**Images:**


*Br. J. Cancer* (1986) **54**, 631–636

### The immunohistochemical detection of lymph node metastases from infiltrating lobular carcinoma of the breast

G. Bussolati[1], P. Gugliotta[1], I. Morra[1], F. Pietribiasi[1] & E. Berardengo[2]

[1]*Department of Human Oncology, University of Turin, 10126 Turin and* [2]*Pathological Anatomy and Histology Service, Ospedale Maggiore di San Giovanni Battista, Turin, Italy.*

**Summary** Immunological markers improve specificity and accuracy of cell detection, therefore it is important to evaluate their usefulness in improving standard histological procedures.

This study investigates whether immunocytochemical techniques increase the accuracy of detection, in axillary lymph nodes, of metastatic cells from infiltrating breast lobular carcinoma (ILC).

Fifty cases of ILC reported to be node-negative were selected. New serial sections were cut from a total of 767 lymph nodes, stained with H&E and tested in immunoperoxidase (ABC procedure) with a conventional anti-Epithelial Membrane Antigen (EMA) serum, with a monoclonal raised against human milk fat globule membranes (HMFG-2) and with a monoclonal against 54 kd keratin. Metastases were detected immunocytochemically in 12 cases (24%); in five of these cases metastatic cells were also visible in serial H&E sections. Monoclonals offered no evident advantage over anti-EMA conventional antiserum. Immunocytochemical positivity alone is not sufficient evidence for metastatic invasion since macrophages occasionally appear EMA- and HMFG-2-positive (probably because of secondary incorporation of the antigen), and so an improvement in the accuracy of breast cancer metastatic cell detection in axillary lymph nodes requires a combined histo-immunological approach.

Prognosis and treatment of breast cancer is heavily influenced by the detection of metastases in lymph nodes. Foci formed by clumps of cells are easily spotted in standard histological sections, but a more difficult task is the detection of metastatic foci of one or few cells spread within a tissue.

In as many as 24% of lymph nodes reported free of metastases by standard histological examination, various authors found metastases when multiple or serial sections were cut (Pickren, 1961; Fisher *et al.*, 1978*b*). Immunological markers also improve the specificity and accuracy of cell detection, therefore it is important to evaluate their usefulness in improving standard histological procedures.

Wells *et al.* (1984) utilizing 3 monoclonal antibodies found micro-metastatic foci in about 20% of lymph nodes from 45 cases of ductal or lobular breast cancer reported negative on conventional histology. On the contrary Sloane *et al.* (1980) reached the conclusion that detection of metastases in lymph nodes was not improved by immunocytochemistry. However they used a polyclonal antiserum against fat globule membranes of human milk.

In this study 50 cases of infiltrating lobular carcinoma of the breast reported to have axillary lymph nodes free of metastases were re-examined to determine whether occult metastases were revealed by a higher number of sections and by immunocytochemical procedures and, in cases where metastatic cells could be detected immunocytochemically, whether a substantial difference in results using monoclonal antibodies and polyclonal antisera was demonstrable.

Cases of infiltrating lobular carcinoma rather than the more common ductal carcinoma were examined, because metastatic spread from the former is difficult to diagnose since neoplastic cells are often 'benign-looking' (Ashton *et al.*, 1975) and tend to fill the sinuses, mimicking sinus histiocytosis (Rosai, 1981), or to invade the lympho-reticular tissue in a lymphoma-like pattern difficult to detect on standard histological sections (McDivitt *et al.*, 1968).

### Materials and methods

The specimens consisted of axillary lymph nodes which had been removed at surgery for infiltrating lobular breast carcinoma (ILC). Fifty cases reported to be free of metastases after routine examination of axillary lymph nodes (a minimum of 10 lymph nodes and 15 on the average having been examined), observed between 1978 and 1983 were selected from the files of our two Institutions and of the S. Anna Gynecological Hospital of Turin according to the following criteria: (a) the follow up was available for a minimum of 2 years, (b) no type of therapy had been administered after the first

Correspondence: G. Bussolati.
Received 18 February 1986; and in revised form 30 May 1986.

© The Macmillan Press Ltd., 1986

operation, (c) the original histological sections (stained with H&E) and the paraffin blocks were available.

The blocks were re-cut and 5 serial sections were obtained on gelatinized slides. The first of the sections was stained with H&E. The other 4 sections were employed for the immunocytochemical technique. The ABC procedure (Hsu *et al.*, 1981) was used, with a minor modification to avoid non specific staining of mast cells (Bussolati & Gugliotta, 1983). Endogenous peroxidase was abolished by the hydrogen peroxide-periodic acid-borohydride sequence (Heyderman & Neville, 1977).

The following markers were employed:

1. Goat anti-Epithelial Membrane Antigen (EMA) from Sera Lab, U.K. (diluted 1:400).
2. Mouse monoclonal antibody against human milk fat globule membrane (HMFG-2) (diluted 1:10), a kind gift from Dr J. Taylor Papadimitriou – Imperial Cancer Research Fund, London. Of the two monoclonals produced in Dr Taylor Papadimitriou's Laboratory, HMFG-2 and not HMFG-1 was used, because the epitope recognized by the former is more frequently expressed in metastatic breast cancer cells (Burchell *et al.*, 1983).
3. Mouse monoclonal antibody against 54 kd human keratin (diluted 1:500) described by Gown & Vogel (1984) and supplied by ENZO Biochem (USA). The type of keratin recognized by this antibody is commonly expressed in all epithelial cells (but not lymphoid cells). In preliminary experiments we found that this monoclonal, although giving better results on alcohol fixed tissues as is the case for all anti-keratin antibodies (Altmannsberger *et al.*, 1981), gives a weak but specific staining of breast cancer metastatic cells also in formalin-fixed tissues.

One of the sections was used as a control, treated with normal mouse serum (diluted 1:50) and then processed with horse anti-mouse biotinylated antibody and the ABC procedure.

In special cases, to identify immunocytochemically positive cells, photographs of the corresponding area of serial H&E stained slides were taken. The slide was then unmounted and processed first with standard treatment to abolish the endogenous peroxidase (which removes both haematoxylin and eosin stains) and then for immunocytochemistry. The morphology of the cells could then be easily recognized and pictures of the same cells, before and after immunocytochemistry, compared.

To identify macrophages, rabbit antisera against chymotrypsin and alpha-1 anti-trypsin were employed (both diluted 1:100; from Dako, Denmark). These enzymes are regarded as macrophage markers (Motoi *et al.*, 1980; Isaacson *et al.*, 1981).

### Results

Metastatic ILC cells gave a strong reaction with a similar distribution with both the anti-EMA serum and HMFG-2 antibody, in one case however a group of metastatic cells was positive only with the former. The staining was mainly localized over the cell surface and in occasional intra-cytoplasmic vacuoles with the typical 'targetoid' pattern (Eusebi *et al.*, 1977). A faint diffuse cytoplasmic staining was also a rather common finding.

The anti-keratin antibody gave a negative reaction in lymphoid cells and a definite, although rather weak, staining of ILC metastatic cells. It was thus better not to rely on the use of this monoclonal for recognizing metastatic cells at low-magnification scanning: this was instead feasible on sections stained with the anti-EMA serum. The two monoclonals on serial sections were a useful control of the results obtained with the anti-EMA serum.

In one case anti-EMA serum markedly stained small cytoplasmic dots in a large number of cells located mostly in the peripheral sinuses. This finding was present in an appreciable number of cells in another 5 cases. These cells were interpreted as macrophages because of the positive staining on serial sections with chymotrypsin and alpha-1 antitrypsin and the oval, clear nucleus with ample, star-like cytoplasm (Figure 1a–c). These cells were totally unreactive with anti-keratin serum.

Occult metastases were detected by the antisera in 12 of the 50 cases examined with a total of 26 positive lymph nodes out of the 767 lymph nodes examined.

Metastatic cells occurred mostly as isolated, non-cohesive cells either in the sinuses or in the diffuse lymphoid tissue. In the sinuses they appeared between histiocytes and occasionally filled the sinuses in a rather compact fashion (Figure 2). In some cases, no or very few epithelial metastatic cells were observed in the sinuses, while positive cells were scattered in a diffuse pattern between lymphoid cells in the cortex and medulla (Figure 3a, b; 4a, b).

Examination of the H&E sections serial to the immunocytochemically stained ones identified cancer cells in 7 nodes (5 cases) out of the 26 positive lymph nodes. An independent observer reached the same conclusion. In one of the 5

**Figure 1** Axillary lymph node from a case of ILC. In the peripheral sinus several cells display cytoplasmic positivity for EMA (a). On serial sections the cells are interpretable as macrophages, both morphologically on H&E slides (b) and because of reactivity with anti-chymotrypsin antiserum (c). ( × 400)

**Figure 2** Axillary lymph node from a case of ILC. Neoplastic EMA-positive cells engulfing the sinuses are detectable at low magnification. On a serial H&E slide, cancer cells could be recognized only where present in clumps; the sparse EMA-positive cells (dark spots on the right and lower areas) were not morphologically identifiable ( × 100).

histologically positive cases, cancer cells were found to be present already in the original slides.

In the other 7 cases, the isolated distribution of the metastatic cells described above seems the main reason for failure to detect them by morphology. This was clearly shown when H&E and peroxidase stained photomicrographs of the same cells were compared (Figure 4a, b). Furthermore, cells of infiltrating lobular carcinoma often show a vesicular inconspicuous nucleus and ample cytoplasm which makes them resemble reactive histiocytic cells.

**Figure 3** Axillary lymph node from a case of ILC diagnosed on H&E slides as free from metastases (a). A serial section stained for EMA (b) shows diffuse infiltration by cancer cells in the cortex and medulla. ( × 100).

**Figure 4** A cortical area of an axillary lymph node from a case of ILC, appearing free of metastatic spread on the H&E section (a). The same section was unmounted and re-stained for EMA (b). A comparison of the two pictures gives evidence of the benign-looking appearance of the cancer cells infiltrating the lymphoid tissue. ( × 250).

The follow up of all 50 cases for a minimum of 2 and a maximum of 7 years (mean 3.5 years) showed that 2 of the 12 (17%) immunocytochemically positive cases, and 7 (2 of whom died) of the 38 (18%) negative cases, had recurrences.

### Discussion

This study focuses on the use of immunocytochemistry to detect metastatic breast cancer cells from infiltrating lobular carcinoma (ILC). In a preliminary study in 10 cases of infiltrating ductal carcinoma originally classified as node negative we could not find any advantage in the use of immunocytochemistry (unpublished observation). Small metastatic foci were detected in 2 cases, but these could be already recognized in serial H&E sections. Sloane & coworkers (1980) using a conventional anti-EMA serum were not able to disclose an advantage of immunocytochemistry over morphology in studying 26 cases of node negative breast cancer patients. Wells *et al.* (1984) stated that in most (but not in all) cases examination of conventionally stained slides adjacent to the immunocytochemically stained ones could reveal to an informed eye the presence of metastatic cells.

The results of this study revealed occult metastatic foci in 24% of the cases of ILC with lymph nodes originally classified as negative. Of these only 10% were also revealed by serial sectioning. These figures lead us to conclude that in contrast with the results of our above mentioned preliminary study and with those of Sloane *et al.* (1980), immunocytochemistry has definite advantages over routine histological examination. An explanation of these contrasting conclusions possibly lies in the fact that, contrary to the more common ductal carcinoma, ILC cells metastasize to lymph nodes and elsewhere (Ashton *et al.*, 1975) in a scattered fashion making their recognition difficult, even more so because of their lack of severe cytological atypia. Considering that ILC cases only account for about 10% of breast cancer histological types and that Sloane's cases were not selected specifically from among ILC series, it seems likely that he observed a majority of cases where metastases could already be detected relatively easily at the original examination and thus immunocytochemistry had little to add. The results of Wells *et al.* (1984) were also different from those of Sloane *et al.* (1984), but the explanation they gave as most likely was that Sloane used polyclonals whereas Wells *et al.*, used monoclonals. We do not agree with this explanation, because in our experience anti-EMA antibodies were seen to be very useful (see below). Wells *et al.*, found that immunocytochemistry revealed occult lymph node metastases in 9% of 33 cases of ductal carcinomas and in 33% of 12 cases of lobular carcinomas. This supports our hypothesis that metastatic foci of ILC cases have a higher risk of passing undetected when only morphology is adopted.

A major interpretative problem is presented by the EMA-reactive material, occasionally present (12% of the cases) in macrophages. This material is probably produced by the primary tumour and secondarily incorporated by macrophages. For diagnostic purposes the finding suggests that immunocytochemistry should be accompanied by histological examination.

Anti-EMA and HMFG2 antibodies showed high specificity for neoplastic mammary epithelium within lymph nodes, with neither having any advantage over the other. The faint staining of plasma cells is a pitfall that can be overcome with nuclear counterstaining (Sloane *et al.*, 1983;

Heyderman *et al.*, 1984). Staining with the anti-keratin antibody was the least evident, probably because the original formalin fixation was detrimental to keratin immunostaining Altmannsberger *et al.*, 1981). Anti-EMA serum is known to stain occasional lymphoid cells (Delsol *et al.*, 1984). For unknown reasons such staining is mainly observed in lymphomas, but in our and in other workers' experience (Sloane *et al.*, 1983) this does not usually happen in reactive lymph nodes. Plasma cells did occasionally show a faint staining of the cell membrane with anti-EMA serum; their nature however could be easily interpreted.

The newly discovered positivity in the lymph nodes of 12 cases was evidently determined by very small foci of metastatic cells that can be defined as micrometastatic because if they had been large foci they would probably have been detected in the original morphological examination. Fisher *et al.* (1978*a*) in agreement with Huvos *et al.* (1971) and with Attiyeh *et al.* (1977) observed that in breast cancer patients in whom micrometastases measured less than 1.3 mm, the survival and treatment rates were similar to those of node-negative patients. Rosen & coworkers (1981) observed, however, that patients with micrometastatic spread in axillary lymph nodes behaved, after a long follow up (twelve years), as high risk patients.

The follow up period and the number of cases studied in our series are both too small for us to reach conclusions on whether immunocytochemically positive lymph node cases previously diagnosed at morphology as negative (therefore probably micrometastatic) carry a worse prognosis than cases that remain negative all along. However in this case series (with its already mentioned limitations) we did not find that the 24% of lymph node positive cases had an increased frequency of recurrences or a shortened survival time.

Having now determined the technical utility of immunocytochemistry we intend studying a large series with long term follow up to determine whether it also improves prognostic accuracy.

Study supported by grants from the AIRC (Milan), the MPI (Rome) and the Italian CNR (Finalized Project 'Oncologia') grant no. 85.02058.44.

The authors are grateful to Dr B. Ghiringhello of S. Anna Gynecological Hospital of Turin for supplying several cases. Prof. P.M. Gullino and Dr M. Mostert, of the University of Turin, kindly reviewed the manuscript.

### References

ALTMANNSBERGER, M., OSBORN, M., HOLSCHER, A., SCHAUER, A. & WEBER, K. (1981). The distribution of keratin type intermediate filaments in human breast cancer: An immunohistological study. *Virchows Arch. (Cell Pathol.)*, **37,** 277.

ASHTON, P.R., HOLLINGSWORTH, A.S. JR & JOHNSTON, W.W. (1975). The cytopathology of metastatic breast cancer. *Acta Cytol.*, **19,** 1.

ATTIYEH, F.F., JENSEN, M., HUVOS, A.G. & FRACCHIA, A. (1977). Axillary micrometastasis and macrometastasis in carcinoma of the breast. *Surg. Gynecol. Obstet.*, **144,** 839.

BURCHELL, J., DURBIN, H. & TAYLOR PAPADIMITRIOU, J. (1983). Complexity of expression of antigenic determinants, recognized by monoclonal antibodies HMFG-1 and HMFG-2, in normal and malignant human mammary epithelial cells. *J. Immunol.*, **131,** 508.

BUSSOLATI, G. & GUGLIOTTA, P. (1983). Nonspecific staining of mast cells by Avidin-Biotin-Peroxidase Complexes (ABC). *J. Histochem. Cytochem.*, **31,** 1419.

DELSOL, G., GATTER, K.C., STEIN, H. & 4 others (1984). Human lymphoid cells express epithelial membrane antigen. Implications for diagnosis of human neoplasms. *Lancet*, **ii,** 1124.

EUSEBI, V., PICH, A., MACCHIORLATTI, E. & BUSSOLATI, G. (1977). Morpho-functional differentiation in lobular carcinoma of the breast. *Histopath.*, **1,** 301.

FISHER, E.R., PALEKAR, A., ROCKETTE, H., REDMOND, C. & FISHER, B. (1978*a*). Pathologic findings from the National Surgical Adjuvant Breast Project (protocol no. 4) V. Significance of axillary nodal micro- and macrometastases. *Cancer*, **42,** 2032.

FISHER, E.R., SWAMIDOSS, S., LEE, C.H. & 3 others (1978*b*). Detection and significance of occult axillary node metastases in patients with invasive breast cancer. *Cancer*, **42,** 2025.

GOWN, A.M. & VOGEL, A.M. (1984). Monoclonal antibodies to human intermediate filament proteins II. Distribution of filament proteins in normal human tissues. *Am . J. Pathol.*, **114,** 309.

HEYDERMAN, E., GRAHAM, R.M., CHAPMAN, D.V., RICHARDSON, T.C. & MCKEE, P.H. (1984). Epithelial markers in primary skin cancer: An immunoperoxidase study of the distribution of epithelial membrane antigen (EMA) and carcinoembryonic antigen (CEA) in 65 primary skin carcinomas. *Histopath.*, **8,** 423.

HEYDERMAN, E. & NEVILLE, A.M. (1977). A shorter immunoperoxidase technique for the demonstration of carcino-embryonic-antigen and other products. *J. Clin. Pathol.*, **30** 138.

HSU, S.M., RAINE, L. FANGER, H. (1981). Use of avidin-biotin-peroxidase complex (ABC) in immunoperoxidase technique. A comparison between ABC and unlabeled antibody (PAP) procedures. *J. Histochem. Cytochem.*, **29**, 577.

HUVOS, A.G., HUTTER, R.V.P. & BERG, J.W. (1971). Significance of axillary macrometastases and micrometastases in mammary cancer. *Ann. Surg.*, **173**, 44.

ISAACSON, P., JONES, D.B., MILLWARD-SADLER, G.H., JUDD, M.A. & PAYNE, S. (1981). Alpha-1-antitrypsin in human macrophages. *J. Clin. Pathol.*, **34**, 982.

McDIVITT, R.W., STEWART, F.W. & BERG, J.W. (1968). Tumors of the breast. Atlas of tumor pathology, 2nd series, fascicle 2. Armed Forces Institute of Pathology: Washington, D.C.

MOTOI, M., STEIN, H. & LENNERT, K. (1980). Demonstration of lysozyme, α1-antichymotrypsin, α1-antitrypsin, albumin and transferrin with the immunoperoxidase method in lymph node cells. *Virchows Arch.* (*Cell Pathol.*), **35**, 73.

PICKREN, J.W. (1961). Significance of occult metastases. A study of breast cancer. *Cancer*, **14**, 1266.

ROSAI, J. (1981). Ackerman's Surgical Pathology. vol 2, p. 1214. The C.V. Mosby Company: St. Louis, Toronto, London.

ROSEN, P.P., SAIGO, P.E., BRAUN, D.W. & 3 others (1981). Axillary micro- and macrometastases in breast cancer: prognostic significance of tumor size. *Ann. Surg.*, **194**, 585.

SLOANE, J.P., HUGHES, F. & ORMEROD, M.G. (1983). An assessment of the value of epithelial membrane antigen and other epithelial markers in solving diagnostic problems in tumour histopathology. *Histochem. J.*, **15**, 645.

SLOANE, J.P., ORMEROD, M.G., IMRIE, S.F. & COOMBES, R.C. (1980). The use of antisera to epithelial membrane antigen in detecting micrometastases in histological sections. *Br. J. Cancer*, **42**, 392.

WELLS, C.A., HERYET, A., BROCHIER, J. GATTER, K.C. & MASON, D.Y. (1984). The immunocytochemical detection of axillary micrometastases in breast cancer. *Br. J. Cancer*, **50**, 193.